# Influence of Waste Glass Powder Addition on the Microstructure and Mechanical Properties of Autoclaved Building Materials

**DOI:** 10.3390/ma15020434

**Published:** 2022-01-07

**Authors:** Wojciech Szudek, Łukasz Gołek, Grzegorz Malata, Zdzisław Pytel

**Affiliations:** Department of Building Materials Technology, Faculty of Materials Science and Ceramics, AGH University of Science and Technology, Al. A. Mickiewicza 30, 30-059 Kraków, Poland; golek@agh.edu.pl (Ł.G.); gmalata@agh.edu.pl (G.M.); pytel@agh.edu.pl (Z.P.)

**Keywords:** autoclaved aerated concrete, sand–lime brick, waste glass powder, waste glass cullet, mineral additive, tobermorite, sustainability

## Abstract

Lime quartz samples in which ground quartz sand was gradually substituted with waste glass powder (GP) were obtained under hydrothermal conditions to determine the influence of GP addition on the microstructure (observed by SEM), phase composition (analyzed by XRD), and compressive strength of autoclaved building materials. An additional series containing analytical grade NaOH and no GP was formed to evaluate the effect of sodium ions on tobermorite formation and its impact on the mechanical properties of the samples. GP addition hindered the formation of tobermorite during autoclaving. Instead, a higher amount of an amorphous and semi-crystalline C–S–H phase formed, leading to the densification of the composite matrix. Nevertheless, tobermorite-like structures were found during both XRD and SEM analyses, proving that the presence of small amounts of Al^3+^ ions allowed, to an extent, for the stabilization of the phase despite the high sodium content. The compressive strength values indicate that the presence of alkali in the system and the resulting formation of additional portions of C–S–H have a beneficial influence on the mechanical properties of autoclaved composites. However, the effect fades with increasing glass powder content which, together with a slight expansion of the samples, suggests that at high sand substitution levels, an alkali–silica reaction takes place.

## 1. Introduction

Waste glass deposited in landfills has been an issue for decades, as its recycling is often problematic due to the fact of chemical contamination or the presence of different coloring ions [1,2]. Over the last couple of years, numerous papers have been dedicated to the use of waste glass powder as a pozzolanic additive in traditional mortars and concretes (e.g., [1,2,3,4,5,6,7,8,9,10]). However, only a few describe the utilization of glass powder in the production of autoclaved building materials [11,12,13]. Meanwhile, the transformation of the European energy sector, aimed at the reduction of CO_2_ emissions, results in a rapidly decreasing supply of fly ash, commonly used in the production of autoclaved aerated concrete (AAC). Moreover, the growing prices of carbon dioxide emission allowances combined with increasing awareness of the importance of climate protection and sustainable development has led to a global search for new alternative supplementary cementitious materials (SCMs), similar to conventional fly ash, that could be used as components of concretes and binders, lowering the consumption of quicklime and Portland cement [14]. Recently described examples include, i.a., ground waste expanded perlite [15,16,17], gaize [18,19], various mine tailings [20,21,22], fluidized bed combustion fly ashes [23,24,25], and municipal waste incinerator bottom ashes [26,27]. However, the widescale utilization potential of many of these additives is limited due to the fact of their low supply or, especially in the case of some ashes, variations in the chemical composition among different batches of the material [27]. On the other hand, waste glass is available worldwide in large quantities. It contains a high amount of reactive, amorphous silica and, depending on the origin, its chemical composition can be relatively stable, especially in the case of packaging glass [1,6].

It has been reported that the high content of alkali and amorphous silica in waste glass makes traditional mortars and concretes with its addition potentially susceptible to alkali-silica reaction (ASR). This reaction produces a highly expansive gel that leads to the formation of cracks within the composite matrix and, ultimately, to the deterioration of its mechanical properties [2,3,8,28,29,30]. However, numerous authors have determined that ASR can be mitigated by grinding the recycled glass to a particle size below 300 µm. Moreover, milling below 100 µm greatly increases the pozzolanic activity of the material which, in turn, leads to an increase in the compressive strength of the composites [2,3,4,6,8,29,30]. Furthermore, Idir et al. [28] determined that the addition of glass powder ground to a grain size below 120 µm allowed for a reduction of the expansion of mortars containing coarse alkali-reactive aggregates by up to 90%. Similar findings were reported for autoclaved samples. According to Walczak et al. [11], the pozzolanic characteristics of glass powder, introduced as a substitute of ground quartz sand in unfoamed samples with a chemical composition corresponding to AAC, allowed for the slight enhancement of their compressive strength. The increase was, however, marginal (2.4%). Stępień et al. [12] replaced quartz sand in autoclaved sand–lime bricks with waste glass sand and observed a gradual increase in compressive strength with the increasing additive content. The materials were, however, considerably different in particle size diameter (0.5–2.0 mm for quartz sand and 80–160 µm for glass powder).

The introduction of mineral additives into the hydrating system results in the presence of the so-called “foreign ions”, which affect the microstructure and phase composition of the hydrothermal reaction products [31]. In the case of waste glass powder, these ions include Na^+^ and Al^3+^. The main hydration products found in AAC and sand–lime bricks are amorphous or a semi-crystalline C–S–H phase and tobermorite. The latter is commonly considered as the main phase responsible for the mechanical strength of autoclaved building materials. Nocuń-Wczelik [32] determined that if the Na_2_O content in the autoclaved sample is lower than 20%, and no detectable sodium–calcium silicate phases were produced. Instead, the crystallization of other calcium silicate hydrates were affected. The presence of Na^+^ ions accelerates the dissolution of the silica-bearing component and the formation of amorphous C–S–H; however, it hinders the transition of C–S–H to tobermorite. At high Na_2_O concentrations, the tobermorite formed is characterized by a highly disordered structure, or it is not present at all. Meanwhile, aluminum ions can be incorporated into the hydrates’ structure both at calcium and silicon sites, which stabilizes the tobermorite. According to the author, the latter of the competitive effects was predominant in the samples where both Na^+^ and Al^3+^ ions were present—for mixtures characterized by a CaO/SiO_2_ molar ratio of 1.00 and containing 10% of Al_2_O_3_ and over 20% of Na_2_O, tobermorite was the main phase present. These results were confirmed by Majdinasab and Yuan [13], who obtained crystalline tobermorite using waste glass cullet as a precursor.

Nevertheless, when using waste glass powder as an additive in autoclaved building materials manufacturing, the presence of sodium ions originating from the dissolving cullet should, at least to some extent, hinder the formation of tobermorite and promote the formation of semi-crystalline or amorphous C–S–H phase. As mentioned by Tian et al. [31], such an effect could positively affect the mechanical strength of the material. Therefore, a study was conducted to determine the influence of waste glass powder addition on the microstructure, phase composition, and compressive strength of autoclaved mixtures containing quicklime and ground quartz sand. An additional series, containing analytical grade NaOH and no glass cullet, was also formed in order to evaluate the hindering effect of sodium ions on tobermorite formation, and its impact on the mechanical properties of the samples.

## 2. Materials and Methods

Samples were obtained through hydrothermal treatment of industrial-grade quicklime (96.4% CaO), ground quartz sand, and ground waste glass powder mixed in different proportions at a water/solid ratio of 1.0 (Table 1). The glass powder was introduced as a substitute for quartz sand in the amounts of 10%, 25%, 50%, and 100%. Its chemical composition is presented in Table 2. For the sample containing 100% of glass powder as an aggregate, the *w*/*s* ratio was reduced to 0.7, and the quicklime content was lowered in order to match the consistency and the CaO/SiO_2_ molar ratio of the reference paste (C/S = 0.83, corresponding to the tobermorite present in ACC). In the case of the GP0Na sample, 1 wt% of Na_2_O (in relation to the dry mass of raw materials) was additionally introduced in the form of sodium hydroxide solution by mixing analytical grade NaOH with slaking water. Dry raw materials were sieved together twice through a 100 µm mesh in order to homogenize them. Subsequently, water was added, and the components were mixed manually for 15 min. Afterwards, 18 mm × 18 mm × 18 mm cubes were formed out of the resulting mixtures, compacted on a vibrating table, and placed in a laboratory oven in sealed containers for 24 h at 80 °C. Next, the samples were demolded and autoclaved for 8 h at 200 °C under a saturated steam pressure of approximately 1.5 MPa. The quartz sand was acquired from a Polish AAC production plant and was characterized by a Blaine specific surface area of 2200 cm^2^/g. The waste glass powder was ground to approximately 4400 cm^2^/g in order to prevent the alkali–silica reaction.

**Figure 1:**
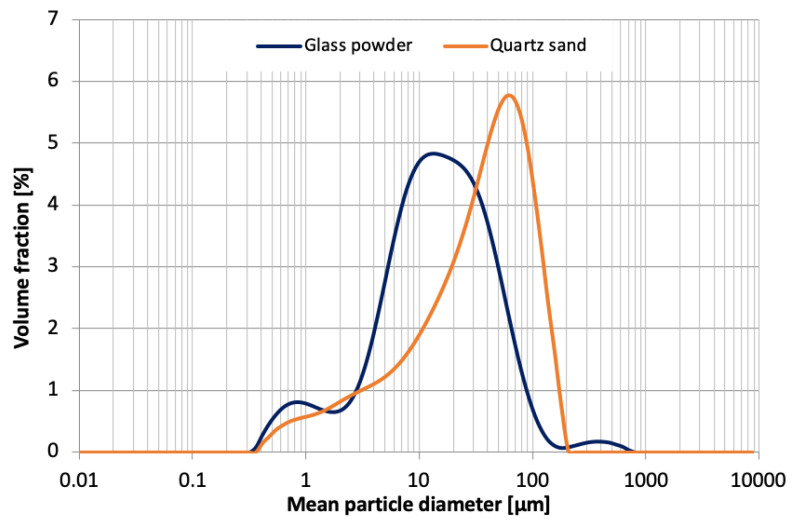
Grain size distribution of ground quartz sand and waste glass powder used in the study.

Grain size distribution of ground quartz sand and waste glass powder (Figure 1) was studied with a Malvern Mastersizer 2000 laser diffractometer using water as a dispersant. The chemical composition of the glass powder was determined using a WDXRF Axios mAX spectrophotometer equipped with a 4 kW Rh anode X-ray tube. XRD analysis was performed using a Philips PW 1050/70 diffractometer (Cu/Ni lamp operating at 35 kV/16 mA, 2θ range of 5–40°, and step size of 0.05°) to determine the phase composition of the autoclaved samples. Diffractograms were analyzed using X’Pert HighScore software (PDF2 database). SEM + EDS observations were carried out on sections and fractures using an FEI Nova Nano SEM 200 microscope in order to evaluate the difference in matrix densification and hydration degree of the samples with and without the addition. Finally, compressive strength of the samples was determined with a Controls Automax 5 universal testing machine to assess the influence of glass powder on the mechanical properties of the composites.

## 3. Results and Discussion

**Figure 2:**
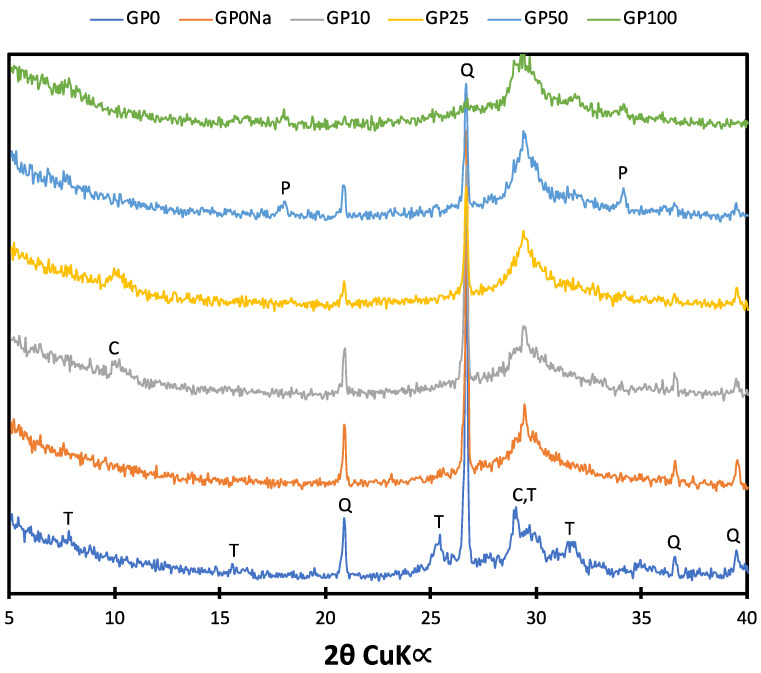
Diffractograms obtained for the autoclaved samples. T: 11Å-tobermorite; Q: quartz; C: calcium silicate hydrates; P: portlandite.

The diffractograms obtained for the autoclaved samples are presented in Figure 2. The XRD pattern of the reference sample revealed the presence of peaks characteristic of unreacted quartz and 11Å-tobermorite. The background elevation in the range of 26–32°2θ was related to the presence of the amorphous and semi-crystalline C–S–H phase. The characteristic tobermorite peaks were not present in the diffractograms of the samples containing NaOH and up to 25% of glass powder, which proves the hindering effect of sodium on the transformation of C–S–H into crystalline products. Traces of the phase were, however, found in the samples containing 50% and 100% of GP, which was probably related to the presence of a small amount of aluminum oxide in the cullet and the stabilizing effect of Al^3+^ ions [32]. Moreover, in the patterns of these two samples, peaks characteristic of portlandite (crystalline calcium hydroxide) were observed. A more distinctive background elevation in the 26–32 °2θ range in the case of samples with both analytical grade NaOH and GP addition lead to the conclusion that due to the high alkali content, a higher amount of amorphous C–S–H phase was produced at the expense of crystalline phases such as tobermorite. The patterns of samples containing the addition of NaOH solution and 10% of waste glass powder were very similar. However, the elevation observed in the angular range of 10–12 °2θ of the GP10 and GP25 diffractograms proves a change in the structure of the semi-crystalline C–S–H phase, most likely due to the incorporation of foreign ions originating from cullet dissolution.

**Figure 3:**
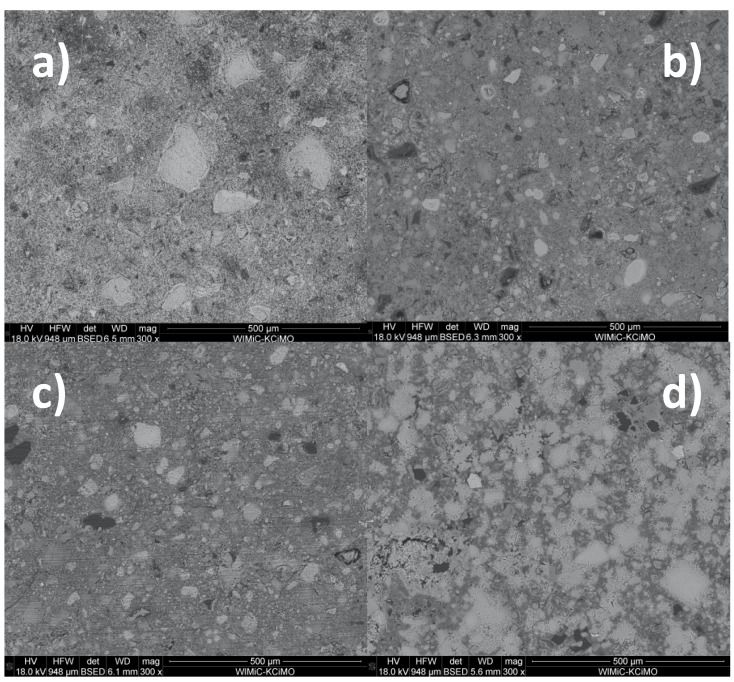
Typical SEM micrographs of the cross-sections of autoclaved samples: (**a**) GP0; (**b**) GP25; (**c**) GP50; (**d**) GP100.

**Figure 4:**
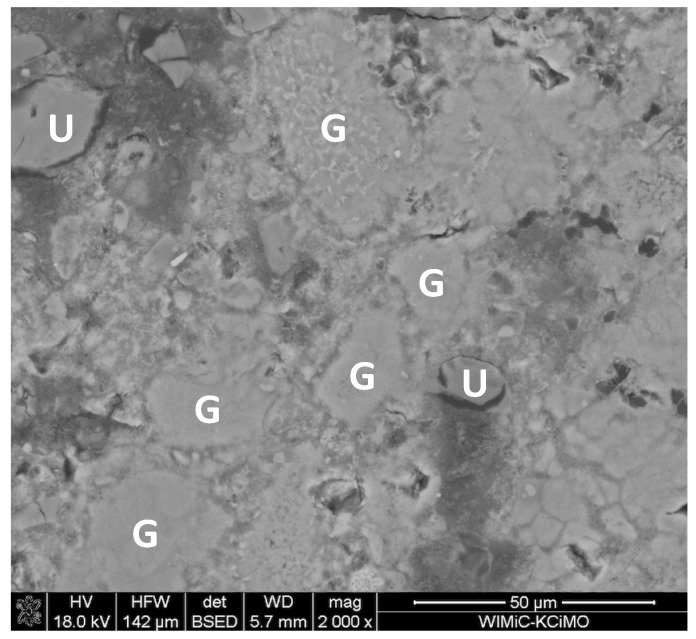
The GP100 sample: (**G**) fully dissolved and (**U**) unreacted glass powder grains.

Typical SEM micrographs of the cross-sections of autoclaved samples are presented in Figure 3. The conclusions drawn from their observations correspond to the results of the XRD analysis: the addition of waste glass powder leads to the densification of the composite matrix due to the formation of additional amounts of the amorphous and semi-crystalline C–S–H phase. The degree of sand grain dissolution in the GP-containing samples was higher than in the reference paste. The glass powder grains were mostly dissolved; therefore, the microstructure of the sample containing 100% cullet as an aggregate was the most packed (Figure 4). Such a result was expected due to the metastable character of the glassy state [2]. However, it should be emphasized that the aggregates differed in specific surface area, which also significantly affects the hydration degree of the samples containing high amounts of waste glass powder.

**Figure 5:**
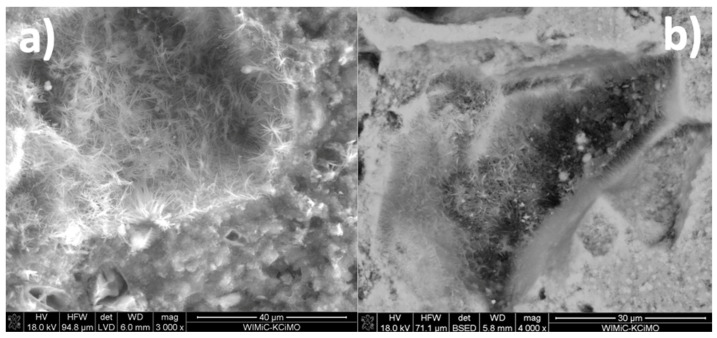
(**a**) Needle-like and (**b**) platelet-like structures found in the GP50 and GP0Na samples.

SEM observations carried out on fractures revealed that apart from the amorphous C–S–H phase, small amounts of fibrous or even tobermorite-like structures were present in all of the samples containing Na^+^ ions (Figure 5). This corresponds to the results of the XRD analysis, where traces of tobermorite were found in the GP50 and GP100 mixtures and suggests that the phase was either present in other samples in quantities below the detection limit of the method (typically 1–2%), or the crystalline forms observed under the microscope were not tobermorite but well-ordered semi-crystalline C–S–H.

**Figure 6:**
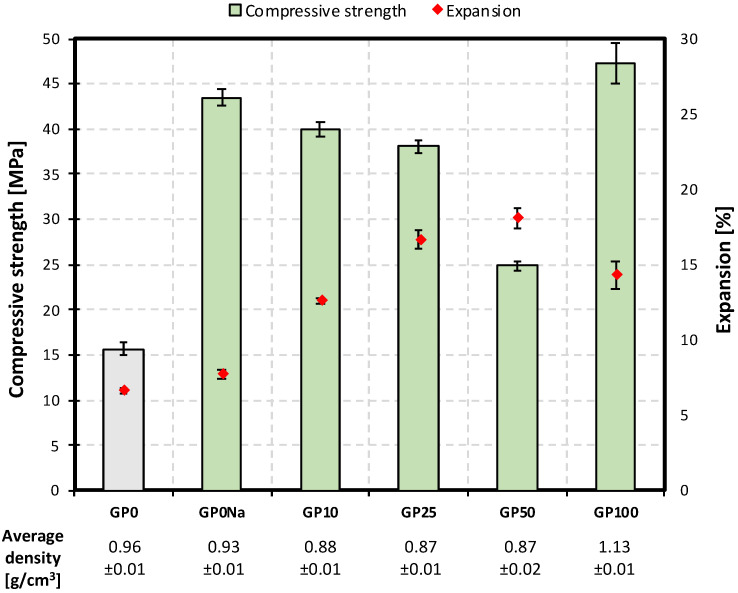
Compressive strength, expansion (relative changes in the linear dimensions of the samples after hydrothermal treatment), and density of autoclaved samples.

**Figure 7:**
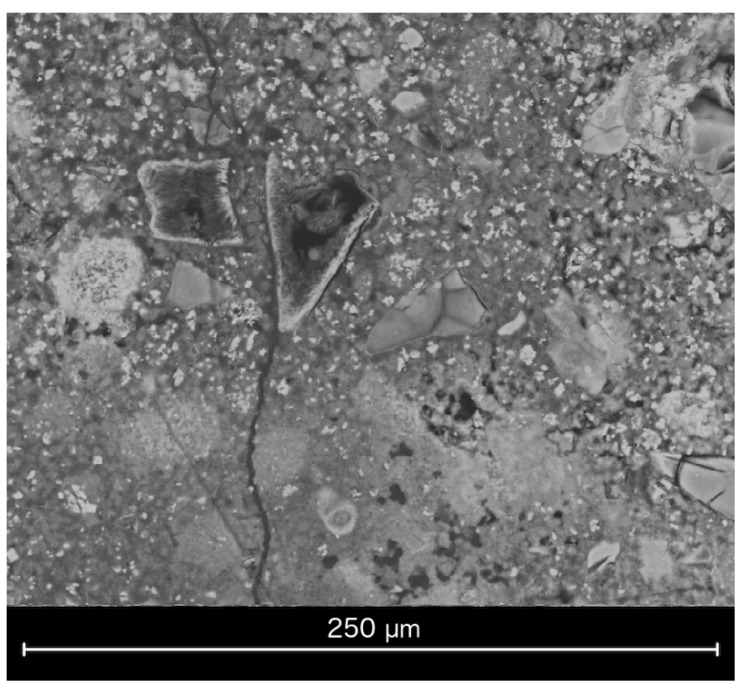
Microcracks present in the cross-section of the GP50 sample.

The results of the compressive strength measurements are presented in Figure 6. A considerable increase in mechanical strength was observed for all of the samples containing glass powder. However, the highest value was recorded for the paste with the lowest GP addition (10%). Increasing the cullet content, despite the fact that the material was characterized by a higher specific surface area and reactivity than quartz sand, resulted in lower compressive strength values, compared to the GP10 sample. The effect was not observed in the case of the sample containing 100% glass powder as the aggregate. On the contrary, its average compressive strength, though improved also by the lowered *w*/*s* ratio (0.7), was the highest out of all of the examined mixtures, which corresponds to the results of SEM observations, where the matrix of the GP100 paste was the most packed and the apparent hydration degree of the aggregate was the highest. This suggests that the decrease in strength was related to the presence of quartz sand together with large amounts of glass powder. SEM images of the GP50 sample, characterized by the lowest average compressive strength out of all of the glass-containing mixtures, revealed the presence of microcracks in the composite matrix (Figure 7), which might suggest that despite the fineness of the waste glass powder, an alkali–silica reaction occurred. The same conclusions can be drawn from the slight expansion observed in the series. However, further research is required to confirm this hypothesis. The density of the samples containing up to 50% of glass powder in the aggregate was slightly lower, compared to the reference paste, which might be the result of a lower pycnometric density of the material itself (2.55 g/cm^3^, compared to 2.65 g/m^3^ for ground sand). The lack of density variations between the GP10, GP25 and GP50 samples can be explained by the increasing content of chemically bound water; however, DTA/TG analysis would have to be performed to confirm it. The slight expansion of the samples also affected their density values. The density of the GP100 sample was considerably higher than any of the other series as a result of its lower *w*/*s* ratio.

The compressive strength of the samples containing the addition of analytical grade NaOH was almost three times higher, compared to the reference, which proves that the formation of an amorphous and semi-crystalline C–S–H phase instead of tobermorite, resulting from the presence of sodium ions in the system, positively impacts the mechanical properties of autoclaved materials. However, it needs to be emphasized that the results were most likely inflated due to the pre-hydration conditions (80 °C, 24 h, and 100% relative humidity). Such treatment was necessary in order for the samples to obtain enough mechanical strength so that they could be demolded. The presence of sodium ions in the system, as mentioned in prior work [32], increases the dissolution rate of the silica-bearing component, especially in the already high pH resulting from the presence of calcium hydroxide. Therefore, the modified samples were hydrated to a higher degree due to the fact of alkali-activation even before they were autoclaved, which definitely contributed to the overall increase in their compressive strength.

## 4. Conclusions

According to the conducted research, the presence of alkali ions, resulting from the introduction of waste glass powder, hinders the formation of tobermorite during hydrothermal treatment of autoclaved building materials. Instead, a higher amount of an amorphous and semi-crystalline C–S–H phase was formed, leading to the densification of the composite matrix. However, traces of tobermorite and tobermorite-like structures were found during both XRD analysis and SEM observations, which leads to the conclusion that the presence of small amounts of Al^3+^ ions allows, to an extent, for the stabilization of the phase even despite the high sodium content, which corresponds to prior research by Nocuń-Wczelik [32]. The results obtained in this study differ from previous works by Walczak et al. [11] and Stępień et al. [12], who reported the presence of considerable amounts of tobermorite in the samples containing large amounts of waste glass powder as a substitute for quartz sand.

The obtained compressive strength values indicate that the presence of alkali in the system and the resulting formation of additional portions of the C–S–H phase have a beneficial influence on the mechanical properties of autoclaved composites. However, the gradual decrease in the degree of the effect with increasing glass powder content, together with the slight expansion of the samples allows to suspect that at high sand substitution levels, an alkali–silica reaction takes place. Further research is required to confirm the hypothesis. An increase in compressive strength was also observed in the case of the sample with analytical grade NaOH addition, which proves that the densification of the matrix, resulting from the introduction of alkali ions, the formation of additional amounts of amorphous and semi-crystalline C–S–H, and the hindering of its transition into crystalline tobermorite allows for the enhancement of the mechanical properties of the composites, despite the lack of the latter. However, it has to be emphasized that the obtained results were inflated by the conditions in which the samples were pre-hydrated.

## Figures and Tables

**Table 1:** Mix proportions of the samples.

Sample Designation	Quicklime (g)	Quartz Sand (g)	Waste Glass Powder (g)	Water (g)	Analytical Grade NaOH (g)	CaO/SiO_2_ Molar Ratio (–)
GP0	64.32	80.0	0.0	144.32	0.0	0.83
GP0Na	64.32	80.0	0.0	144.32	1.86	0.83
GP10	64.32	72.0	8.0	144.32	0.0	0.87
GP25	64.32	60.0	20.0	144.32	0.0	0.93
GP50	64.32	40.0	40.0	144.32	0.0	1.05
GP100	35.60	0.0	80.0	80.92	0.0	0.83

**Table 2:** Chemical composition of the waste glass powder and quicklime.

Material	Compound:	SiO_2_	CaO	Al_2_O_3_	MgO	Na_2_O	K_2_O
Glass powder	Content:	69.0	10.5	2.3	1.4	15.2	0.4
Quicklime		1.0	96.4	0.4	1.6	0.0	0.0

## Data Availability

The data presented in this study are available upon request from the corresponding author.
